# Fabrication of Porous Collagen Scaffolds Containing Embedded Channels with Collagen Membrane Linings

**DOI:** 10.3390/mi15081031

**Published:** 2024-08-14

**Authors:** Neda Fakhri, Arezoo Khalili, Terry Sachlos, Pouya Rezai

**Affiliations:** Department of Mechanical Engineering, York University, Toronto, ON M3J 1P3, Canada

**Keywords:** collagen scaffold, basement membrane, interstitial matrix, vesicle, enclosed microchannel

## Abstract

Tissues and organs contain an extracellular matrix (ECM). In the case of blood vessels, endothelium cells are anchored to a specialized basement membrane (BM) embedded inside the interstitial matrix (IM). We introduce a multi-structural collagen-based scaffold with embedded microchannels that mimics in vivo structures within vessels. Our scaffold consists of two parts, each containing two collagen layers, i.e., a 3D porous collagen layer analogous to IM lined with a thin 2D collagen film resembling the BM. Enclosed microchannels were fabricated using contact microprinting. Microchannel test structures with different sizes ranging from 300 to 800 µm were examined for their fabrication reproducibility. The heights and perimeters of the fabricated microchannels were ~20% less than their corresponding values in the replication PDMS mold; however, microchannel widths were significantly closer to their replica dimensions. The stiffness, permeability, and pore size properties of the 2D and 3D collagen layers were measured. The permeability of the 2D collagen film was negligible, making it suitable for mimicking the BM of large blood vessels. A leakage test at various volumetric flow rates applied to the microchannels showed no discharge, thereby verifying the reliability of the proposed integrated 2D/3D collagen parts and the contact printing method used for bonding them in the scaffold. In the future, multi-cell culturing will be performed within the 3D porous collagen and against the 2D membrane inside the microchannel, hence preparing this scaffold for studying a variety of blood vessel–tissue interfaces. Also, thicker collagen scaffold tissues will be fabricated by stacking several layers of the proposed scaffold.

## 1. Introduction

Tissue engineering is a multidisciplinary field for repairing, regenerating, and or transplanting injured body organs and tissues [1]. A variety of natural (e.g., collagen [2], gelatin [3], alginate [3], and chitosan [4]) and synthetic (e.g., polycaprolactone [5], poly lactic acid [6], and poly vinyl alcohol [7]) materials have been used to fabricate engineered tissues. Although synthetic materials offer a wide variety of properties and are adaptable to different fabrication processes, they usually lack biological relevance to corresponding native tissues [1,2]. Natural materials, however, can provide biomimetic structures for cell culturing and proliferation. For example, collagen, the most abundant protein in the extracellular matrix (ECM), has been used as an appropriate candidate for various tissue engineering applications [3,7] due to its inherent binding motif that promote cell adhesion.

Properly engineering the properties and structures of collagen ECMs and embedding channels in them to simulate vascularization has been technically challenging [4,8]. Moreover, although all ECMs are composed of proteins, polysaccharides, and water, the composition, mechanical properties, structure, and topology of ECMs for each tissue are different. The unique ECM of each tissue evolves through tissue development in a dynamic and reciprocal cross-talk between the cells and their biochemical and biomechanical microenvironments [9].

ECMs consist of two distinct components: (i) the interstitial matrix (IM) and (ii) the basement membrane (BM). IMs are 3D porous networks of heterogeneous textures that have both structural and signaling functions [10]. The BM is a thin, dense, sheet-like layer of ECM used by epithelial/endothelial cells for anchorage. BMs have a wide variety of functions, such as acting as a substrate for cell adhesion and migration, separating tissues and acting as a barrier to prevent the transmigration of most cells (except for leucocytes and stem cells) [11], shaping tissues through their composition [12], and acting as a reservoir for growth factors [13].

The developed collagen-based scaffolds in the literature do not contain all of the abovementioned elements of the corresponding in vivo tissues. A number of vascularized collagen-based tissue-engineered scaffolds have been reported, but they contain either an IM [14,15,16,17] or a BM [18,19,20,21,22]. Incorporating the BM into regenerated tissues and engineering its properties makes these tissues more physiologically relevant to the corresponding in vivo tissues and, eventually, the corresponding organs [23,24].

Transwell inserts are the most common tools used to study the function of the BM in co-culture systems. By using transwell inserts, cells may be cultured in a manner that allows them to communicate with one another while physically isolated. Using transwell inserts, however, does not allow flowing media over the growing cells. In several studies, microstructures (e.g., micropillars and microgrooves) have been used to perform some of the roles of basement membranes in a co-culture system [25,26]. In these systems, vertical and horizontal compartment microfluidics are commonly used to mimic the role of the IM. However, these microfluidic designs lack the 3D IM, as cell culturing is implemented on the surface of 2D microfluidic channels coated with biological materials such as proteins.

The fabrication of collagen-based engineered tissues has been reported in several studies, wherein their corresponding designs contain BM analogs (i.e., 2D films) attached to 3D scaffolds. These tissues were fabricated by laminating cast 2D collagen films to 3D scaffolds [27,28,29]. None of these studies incorporated microchannels in their designs, so they do not have any flowing media through their engineered tissues. Recently, a 2D/3D silk-based scaffold containing a straight conduit-shaped microchannel was fabricated by using a stainless-steel template rod during scaffold fabrication and removing this rod at the end of the process [30]. However, this interesting method is limited in terms of the fabrication of more complex microchannels with branches and changing dimensions.

In this paper, we report a physiologically relevant collagen-based scaffold with vesicle-like microchannels. This scaffold comprises a 3D porous IM collagen layer with embedded microchannels representing vessels, in which the interior surface of the channel is separated from the 3D porous IM by a 2D collagen BM. Two collagen-based substrates, one plain and one with microchannels patterned on it, are replica molded and then bonded together using contact printing on the collagen. Each substrate contains two parts: (i) the relatively dense BM fabricated by drying the collagen at room temperature, and (ii) the porous 3D collagen back-layer fabricated with freeze drying. The bonded scaffolds withstand flow rates as high as 10 mL/min in leakage tests. The integration of a 2D BM and 3D IM in a collagen scaffold not only results in biological relevance but also preserves the integrity of the channels while inhibiting convective leakage of the flowing medium from the microchannel into the ECM. This is important because fluid exchange between the blood vessels and the surrounding tissues is mostly non-convective and based on a selective mechanism controlled by endothelial cells in response to surrounding stimuli. The proposed scaffold can have a wide application in cell and tissue studies and toxicity assessment in drug discovery. For example, it can serve as an ECM in which various types of cells such as endothelial cells [31] and mesenchymal stem cells [32] can be cultured to model different tissues and how they interface with blood vessels.

## 2. Materials and Methods

**Figure 1:**
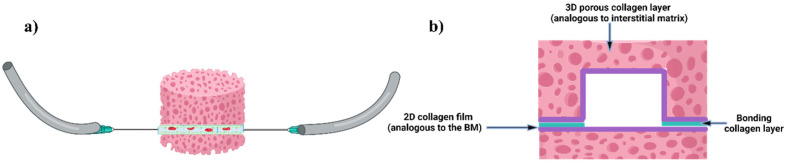
Schematic diagrams of the proposed collagen scaffold simulating 3D porous tissues with embedded microchannels that have inner-wall 2D membrane linings. (**a**) The final scaffold with an embedded microchannel connected to inlet and outlet tubing. (**b**) Close-up cross-sectional view of the microchannel with the 2D BM lining embedded inside the 3D porous IM, which was formed by bonding two collagen layers. (Created with BioRender.com.)

We fabricated a collagen scaffold containing three main components: (i) 3D porous collagen foam resembling the IM, (ii) a 2D collagen film resembling the BM, and (iii) microchannels embedded within the scaffold to be used in the future as blood vessels (Figure 1). The microchannel was thoroughly lined with the 2D collagen BM and fully enclosed within the 3D porous IM. Microchannels were designed with square-shaped cross-sections in which the width and height of the channels varied in the range of 300–1500 μm, falling in the range of medium arteries and veins [33].

### 2.1. Fabrication of Collagen Layers and Scaffolds with Microchannels

**Figure 2:**
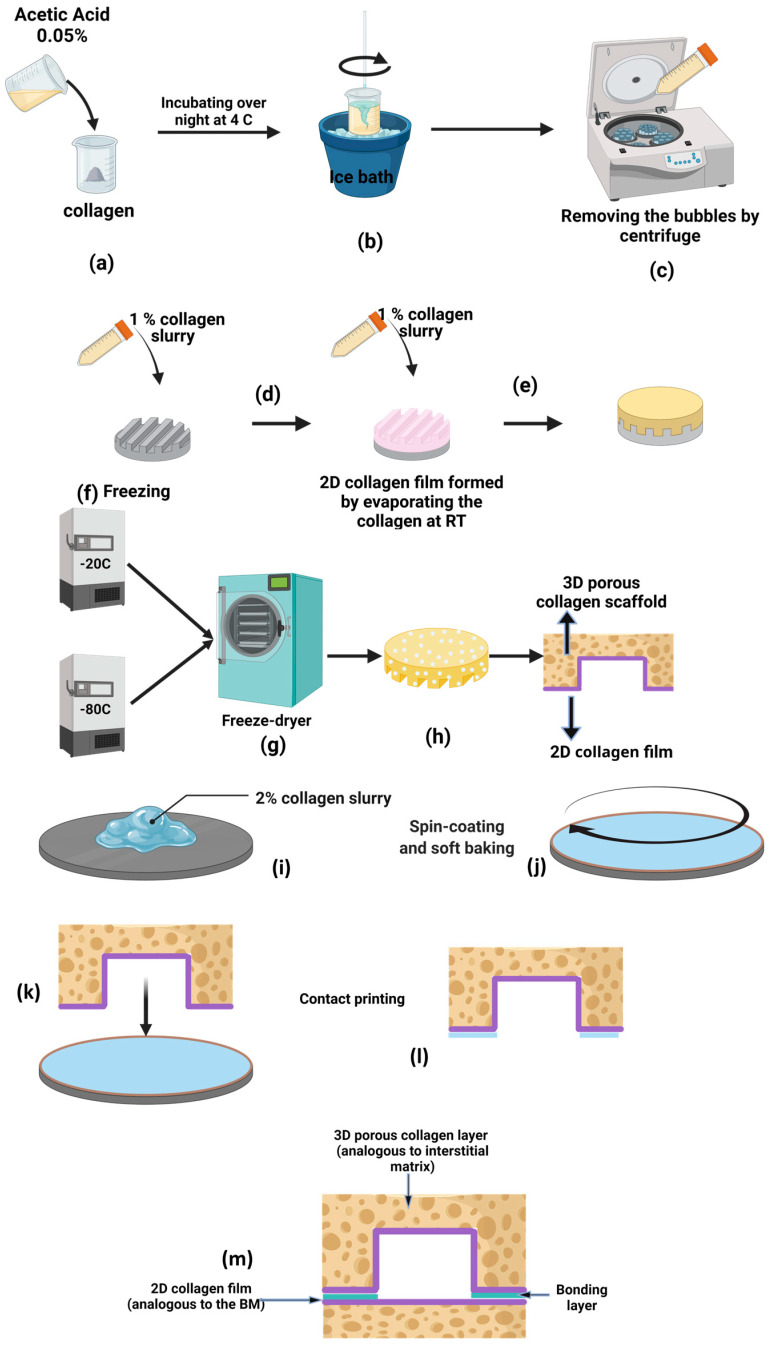
A schematic diagram showing (**a**–**j**) the fabrication procedure of the collagen microchannels and (**k**–**m**) the contact printing method for bonding the patterned collagen with a second flat collagen layer. (Created with BioRender.com).

Collagen slurry was casted on PDMS-based replication molds, then dried at room temperature or inside a freeze-dryer, as shown schematically in Figure 2. A positive PDMS replica mold containing 6 parallel channels with square cross-section sizes ranging from 300 to 1500 µm was first fabricated. PDMS elastomer and curing agent (Sylgard 184 kit from Dow Corning, Midland, MI, USA) were mixed at a 10:1 ratio. The mixture was degassed in a vacuum chamber and poured on a 3D-printed negative mold to make the positive PDMS replica. The degassed PDMS mixture was then cured on a hot plate for 2 h at 80 °C before we peeled it off from the mold. To fabricate the collagen-based microchannel test structures in Figure 2, first, a suspension of 1% *w*/*v* concentration collagen was prepared by mixing 1 g of fibrillar collagen type I from bovine Achilles tendon (Sigma Aldrich, Burlington, MA, USA) with 0.05 mol/L acetic acid (Figure 2a). The resulting collagen slurry was then incubated overnight at 4 °C and agitated using a conventional blender (on an ice bath to keep the temperature low) to become homogenous (Figure 2b). The resulting bubbles of the collagen slurry were removed by centrifuging it at a speed of 400× *g* (Figure 2c). In the next step, the collagen slurry was poured over the patterned PDMS mold and left at room temperature so that the water in the collagen slurry evaporated and a 2D collagen film was formed (Figure 2d). In step e, a freshly prepared collagen slurry was poured on top of the 2D collagen film on the PDMS mold and frozen at a fixed temperature of either −20 °C or −80 °C (Figure 2e,f). Then, the frozen collagen was freeze-dried (Figure 2g), resulting in an integrated 3D porous collagen layer (IM) attached to a dense, 2D collagen BM (Figure 2h). The fabricated collagen scaffold was cut using a diamond wire saw (DWS 100, Diamond Wire Tec, Weinheim, Germany) to obtain a cross-sectional view of the fabricated test structures.

The resulting width, height, and perimeter of each fabricated test structure channel were then compared with the corresponding dimensions of this channel in the PDMS replica. This comparison was helpful for determining the behavior of collagen when it is used in the fabrication of microchannels. Similarly, single microchannels containing 2D collagen BMs and 3D collagen IMs were fabricated using the approach shown in Figure 2a–h. A second non-patterned collagen substrate was also fabricated following the same steps to seal the top patterned layer. The top and bottom layers were then bonded using the contact printing method shown in Figure 2i–k. In summary, a layer of 2% collagen slurry was spin-coated at 1500 rpm for 15 s to form a uniform layer of collagen on a glass substrate (Figure 2j). The scaffold containing the microchannel patterns was then contacted with the layer of spin-coated collagen (Figure 2k) and bonded to the plain collagen layer to seal the channel (Figure 2m). Finally, the bonded assembly was left at room temperature so that the water would evaporate.

### 2.2. Mechanical Properties

To determine the mechanical properties of the 3D porous collagen and 2D collagen film, a tensile test was performed using a TA Discovery HR-2 hybrid rheometer. Tensile stress–strain curves were obtained for the fabricated collagen scaffolds, allowing us to evaluate whether the resulting curves were j-shaped, similar to the soft tissues in the body, and to compare the stiffness of the resulting engineered tissues at low strains (i.e., the linear part of the stress–strain curve) with different tissues in the human’s body. Also, the slope of the curve was measured to determine the stiffness of the samples at desired strain ranges. A strain rate (i.e., the time derivative of strain) of 0.005 s^−1^ was used for all of the tensile tests and the stress–strain curves were extracted at two different states of the collagen: dried and hydrated. To make the collagen scaffolds wet, water was introduced into the collagen scaffold when the scaffold was fixed between two clamps of the rheometer. Conducting experiments for wet collagen is interesting because the tissues eventually come into contact with fluids. The behavior of the materials in these situations help us to compare the fabricated collagens with the corresponding tissues in the body.

### 2.3. Micro and Macro Structural Properties

The macroscopic structure of the scaffold (e.g., the widths and heights of the channels, the perimeter of each channel, the cross-section area of the microchannels, and the pore wall thicknesses) was assessed based on optical images of the collagen scaffolds taken using an upright Leica microscope (Stereomicroscope Leica MZ10F, Singapore) with a CMOS monochrome camera (GS3-U3-51S5M-C, Teledyne Flir, Canada). Then, MATLAB (R2021a) codes were developed to measure the above-mentioned macroscopic properties of the scaffold (see the details of the image analysis steps in Figures S1–S5 in the Supplementary Materials).

The microscopic structure of the scaffold (i.e., pore size) was investigated using images acquired from Scanning Electron Microscopy (SEM) analyses (Quanta 3D FEG Thermofisher, USA). Then, the pore size image analysis algorithm from Rabbani et al. was used to characterize and quantify pore size and the pore distribution in the scaffolds [34].

### 2.4. Water Permeability

A test was conducted to investigate the permeability of the 3D porous collagen and the 2D collagen film. Three-dimensional collagen disks (1% *w*/*v*) were fabricated at −20 °C with a diameter and thickness of 11 mm and 10 mm, respectively. Two-dimensional collagen films were fabricated by air drying at room temperature with a thickness and diameter of 50 μm and 11 mm, respectively. We added 2 mL of water over the collagen layers (either the 3D collagen or the 2D film), and recorded the time required for the entire water to pass through the layers. Using the recorded time, the permeability of the layers could be quantified using Darcy’s equation. Prior to performing these experiments, we sufficiently hydrated the collagens so that almost all of the water passed through the layers.

### 2.5. Leak Test

A leak test was performed to evaluate the performance of the embedded collagen channels using the setup shown in Figure S6 in the Supplementary Materials. Two needles were attached to the fabricated collagen scaffolds with microchannels to allow flowing water into the channels (Figure S6b). Prior to this experiment, the collagen scaffolds were submerged in water to mimic the in vivo conditions. The test was conducted in two different models: one where the scaffold comprised a channel lined with 2D collagen film, and one where it did not (Figures S6c and S6d, respectively). In this test, 10 mL of water was passed through the microchannel at different flow rates of 0.005, 0.01, 0.1, 0.5, 1, 5, 10, and 25 mL/min. To quantify the potential leakage of media flowing through the scaffold, we measured the collected volume of water at the outlet at the end of each experiment. Comparing the volumetric flow rate of water at the inlet and outlet, we could calculate the flow rate of water that penetrated the collagen scaffolds.

## 3. Results and Discussions

We first present the results associated with the properties of 2D collagen films and 3D collagen layers, which were later used in the fabrication of the integrated 2D/3D scaffolds with embedded microchannels. Next, the fabrication of the studied microchannel structures with various cross-sectional sizes is investigated to assess the correlation between channel sizes in the master mold and those in the collagen scaffold. Finally, leakage test results for the embedded microchannels are presented to assess the functionality of the proposed collagen scaffold when exposed to media flowing inside the channel.

### 3.1. Two-Dimensional Collagen Film Properties

**Figure 3:**
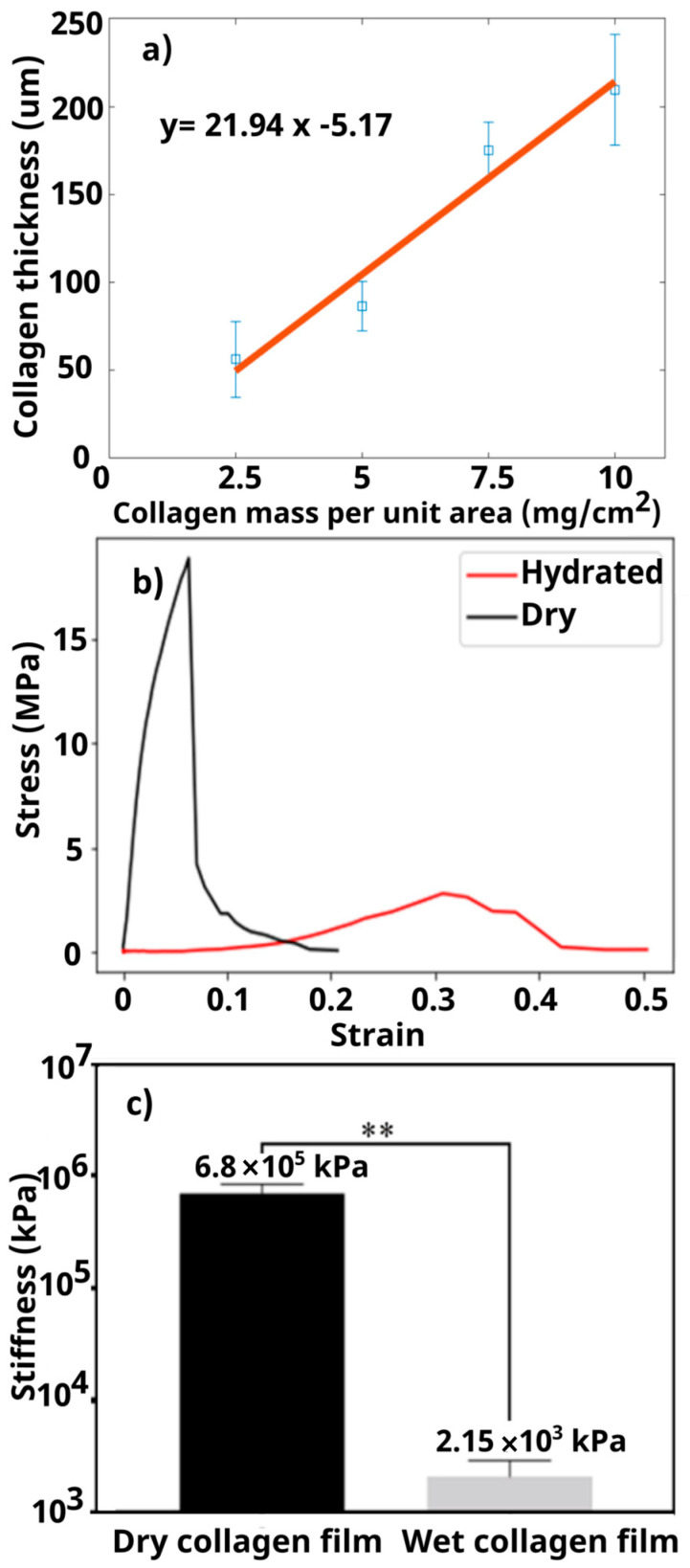
Properties of the 2D collagen film. (**a**) Thickness of the 2D collagen film corresponding to different mass values of collagen per unit area used for fabrication. A solid red line is fitted to the experimental results to assess the linearity of the collagen’s response. (**b**) Tensile stress vs. strain curves for 2D collagen films in dry and hydrated states. (**c**) The stiffness of the 2D collagen films under low strains for the dry and hydrated states. The *p*-value is less than 0.01 (**) for n = 3 data in each group. Error bars show the standard deviations (SD) of the experiments in three trials.

Following the steps demonstrated in Figure 2a–d and using different amounts of collagen, 2D collagen films with various thicknesses were fabricated. The collagen film thickness was controlled based on the total collagen mass used per surface area. As shown in Figure 3a, by using 2.5–10 mg/cm^2^ of collagen slurry, membranes with thicknesses in the range of 56.2 ± 21 µm to 209.5 ± 31 µm could be fabricated, and a linear relationship (R^2^ = 0.96) between the amount of collagen used (x) and the resulting film thickness (y) was obtained (y = 21.94x − 5.17). Liu et al. obtained collagen thickness values of 5 µm to 25 µm, but they did not specify the amount of collagen used per unit area [35]. Further, Wolf et al. fabricated collagen-based films with thickness values ranging from ~35 µm to 180 µm using different collagen masses per unit area ranging from ~6.7 to 23.5 mg/cm^2^ [36]. We acknowledge that the thickness of our collagen membranes is larger than that of a real BM (which is 500 nm in the aorta [37]) because of the handling issues associated with thinner films (i.e., having low mechanical properties and being very brittle). For fabricating collagen scaffolds with microchannels in the following sections, we used a volume of 5 mg/cm^2^ as it was easier to handle during the fabrication process.

The mechanical properties of collagen film as an analog to the BM can play an important role in the behavior of the cultured cells [38]. Cells can behave in a different manner when cultured on collagen films with different stiffness values. Stress–strain curves for two series of experiments involving dry and wet collagen films are shown in Figure 3b. The 2D collagen film shows a brittle behavior in the dry state, meaning that it does not show plasticity (deformation) before rupture. However, when the collagen is hydrated, it shows an entirely different behavior. The stiffness of the hydrated collagen is significantly lower than that of the dry film (Figure 3c), but the hydrated scaffold shows ductile behavior, meaning that it plasticizes and elongates before the rupture, which is attributed to the presence of water inside the hydrated film [39]. The reported stiffness values in Figure 3c are the slope of the stress–strain curves in Figure 3b at low strains. The corresponding stiffness of the in vivo BMs at low strains is reported to be between 2 and 5 MPa for renal tubules and venules [40], which is similar to the stiffness of our hydrated collagen films at small strains.

### 3.2. Three-Dimensional Porous Collagen Properties

**Figure 4:**
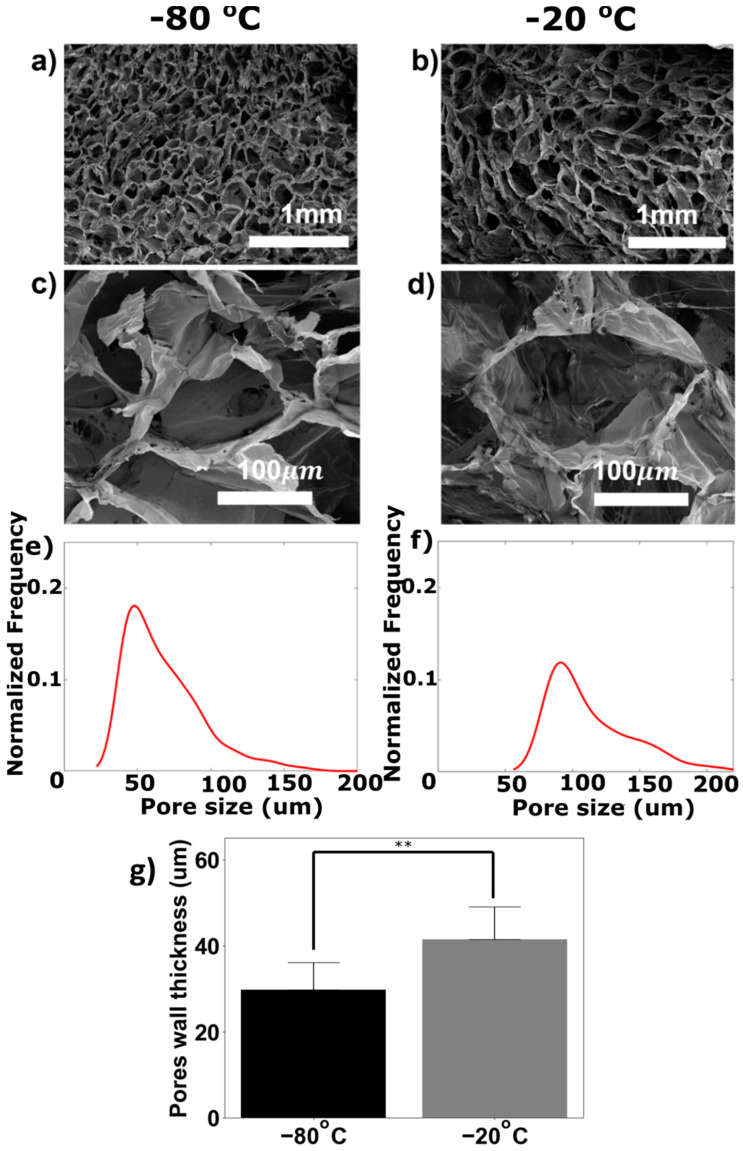
SEM images of 3D porous collagen scaffolds and the corresponding pore size distributions. Porous collagen scaffolds were fabricated using freezing temperatures of (**a**) −80 °C and (**b**) −20 °C; scale bar: 1 mm. SEM images of a single pore in porous collagen fabricated using freezing temperatures of (**c**) −80 °C and (**d**) −20 °C; scale bar: 100 μm. Pore size distribution for collagen scaffolds fabricated using freezing temperatures of (**e**) −80 °C and (**f**) −20 °C. (**g**) Bar plot for the average pore wall thicknesses for fabricated collagens using different freezing temperatures (**: *p*-value < 0.01).

The properties of collagen scaffolds are important as they affect the fate of the cells that are cultured on the resulting engineered tissue. For example, it is important to quantify the pore size of the collagen, because it affects the diffusion and convection of nutrients and water through the porous scaffold. We fabricated 3D porous collagens using two different freezing temperatures to evaluate the appropriate freezing temperature for the fabrication of integrated 2D/3D collagen scaffolds with embedded microchannels. Figure 4a,b show the SEM images for 3D porous collagens fabricated using freezing temperatures of −80 °C and −20 °C, respectively. Further, close-ups of the images in Figure 4a,b are shown in Figure 4c,d, respectively, to compare the pore sizes of the corresponding collagen scaffolds. As shown, a freezing temperature of −20 °C resulted in larger pore sizes, which can be attributed to the formation of larger ice crystals at the freezing temperature of −20 °C compared to at −80 °C. This result is in agreement with the literature reporting a decrease in the average size of the scaffold pores when decreasing the freezing temperature [41,42]. To quantify the sizes of the collagen scaffold pores, image analysis was conducted on SEM images to extract the pore sizes, pore distribution, and pore wall thickness of the collagen scaffolds. Figure 4e,f show the corresponding pore size distributions of the collagen scaffolds fabricated using freezing temperatures of −80 °C and −20 °C, respectively.

The average pore size for the collagen fabricated using a freezing temperature of −80 °C was 67.9 μm, which was less than the 113.1 μm pore size observed at the freezing temperature of −20 °C. O’Brien et al. obtained a pore size of 121 μm for their scaffolds using a freezing temperature of −20 °C at a lower concentration of collagen slurry (i.e., 0.5%) [41]. Faraj et al. obtained an average pore size of 89 μm for their collagen scaffolds using a freezing temperature of −20 °C and a concentration of collagen slurry (i.e., 0.9%) similar to the concentration of collagen slurry used in this research (i.e., 1%) [42]. Further, they obtained an average pore size of 42 μm for their collagen scaffolds fabricated at a freezing temperature of −80 °C. The differences between the pore sizes of the scaffolds in different studies can be attributed to the slightly different protocols used for the fabrication of the collagen. This difference could also be attributed to variations in the source of the collagen that was used for scaffold fabrication. Although the collagen samples in these studies were all sourced from bovine tendon, subtle differences in the animal sources could have influenced the properties of the collagen scaffold pore size. Also, the use of different freezing rates even with a similar final freezing temperature could have a significant effect on the pore size of the scaffold.

Further, it has been reported that the thickness of the fibers in the structure of implanted tissues influences the adhesion properties of the mesenchymal stem cells [43]. Using the image analysis described in the Supplementary Materials, we measured the thickness of the collagen fibers (pore walls) for collagens fabricated using two freezing temperatures. The collagen fibers’ thickness was expected to be higher at a freezing temperature of −20 °C. The average thickness of the collagen fibers was obtained using ten different pore wall thicknesses for each SEM image (Figure 4g). It was significantly larger than the corresponding average wall thickness of collagen pores generated at the freezing temperature of −80 °C. This trend is consistent with the increasing trend reported by Wahl et al. upon switching the freezing temperature from −80 °C to −20 °C [44].

**Figure 5:**
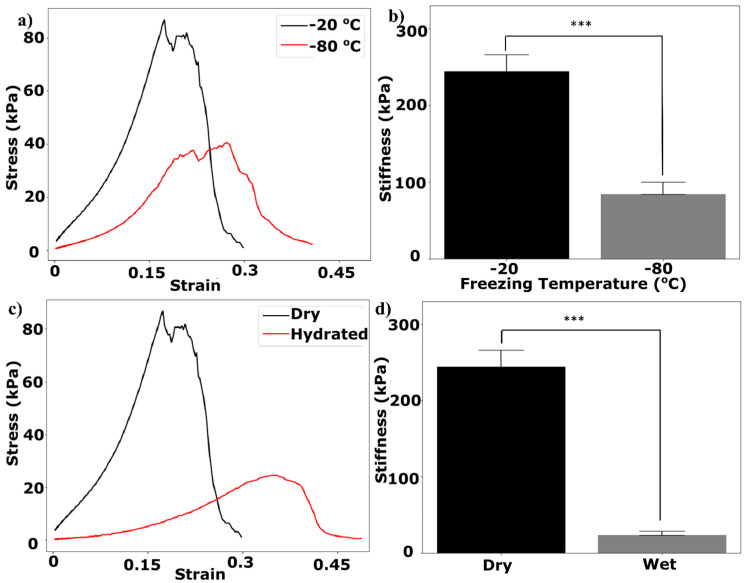
Mechanical properties of dry and hydrated collagen scaffolds fabricated using freezing temperatures of −20 and −80 °C: (**a**) tensile stress–strain curves, (**b**) stiffness under low strain, (**c**) tensile-stress curves for collagen scaffolds fabricated using a −20 °C freezing temperature, and (**d**) stiffness under low strain for dry and hydrated collagen scaffolds based on the curves in panel (**c**). ***: *p*-value < 0.001 for n = 3 data in each group. Error bars show the standard deviations (SD) of the experiments in three trials.

The mechanical properties of 3D porous collagen scaffolds are reported in Figure 5a to enable us to compare their values with the corresponding values found in other studies. Figure 5b shows a comparison between the Young’s modulus values of collagen scaffolds fabricated using the two freezing temperatures of −20 °C and −80 °C. The stiffness of the collagen scaffold was significantly higher at −20 °C than the stiffness of the collagen scaffolds fabricated at a freezing temperature of −80 °C which matches again with the literature [44]. This is mainly attributed to the larger fiber thicknesses of collagen scaffolds at −20 °C. At higher freezing temperatures, more collagen is expelled into the interstices, and thicker collagen pore walls are created, leading to a higher Young’s modulus [44].

Since the collagen scaffold will eventually be used for cell culture purposes and the scaffold will be hydrated, it is important to determine the mechanical properties of collagen in such conditions. According to previous studies, stiffness should decrease when the collagen is hydrated [39]. As shown in Figure 5c,d, the stiffness of the collagen scaffolds fabricated using a freezing temperature of −20 °C decreased significantly when it was hydrated as compared to situations where the collagen scaffolds were dry. The Young’s modulus of the hydrated scaffold in our study was 23.8 MPa and similar to the corresponding value reported by Ryan and O’Brien for their scaffolds fabricated using a relatively similar fabrication approach [45]. Decreasing stiffness for hydrated collagen is attributed to the existence of water molecules among the collagen fibrils decreasing the required stresses for collagen to stretch [39]. As shown in Figure 5c, hydrated collagen scaffold fracture occurs at higher strains (i.e., when there is more ductility). This increase in ductility can be attributed to the existence of water molecules providing additional space for the collagen fibrils to elongate [39].

Note that a freezing temperature of −20 °C was used for the 3D porous collagen scaffold fabrications in the rest of this study. This is because larger pore sizes of the collagen scaffolds fabricated at freezing temperature of −20 °C have been reported to be suitable for cell culture in terms of the cell seeding efficiency and spatial cell distribution within the scaffold [46].

### 3.3. Fabrication and Characterization of Open Microchannels with 2D Membrane Lining in 3D Porous Collagen

**Figure 6:**
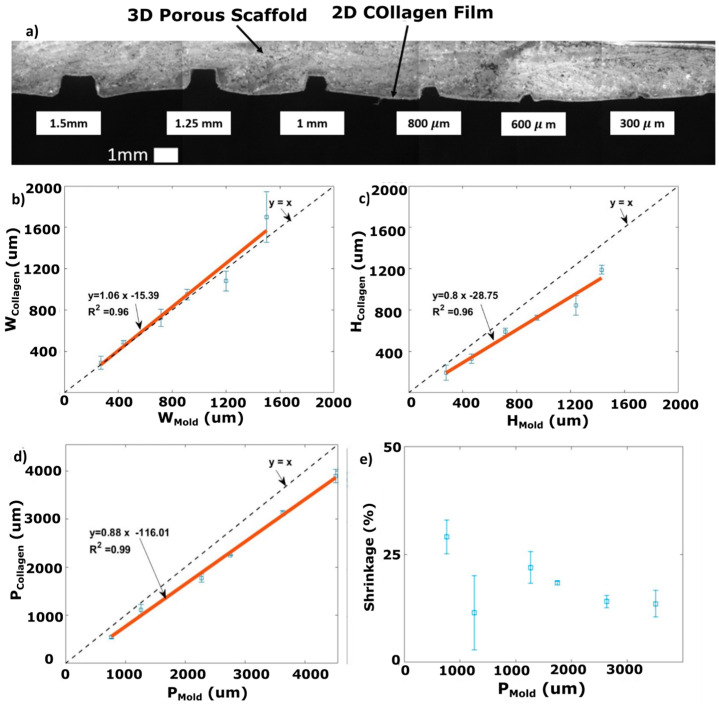
Collagen microchannels with different sizes and comparison between channel sizes in the collagen and channel sizes in the master mold. (**a**) Cross-sectional view of six parallel test microchannels ranging from 300 μm to 1.5 mm in width and height; the respective plots show (**b**) the width (W_Collagen_), (**c**) the height (H_Collagen_), and (**d**) the perimeter (P_Collagen_) of collagen microchannels versus the corresponding dimensions of their PDMS replica master molds; (**e**) shrinkage of the collagen channels based on changes in the perimeter of the collagen compared to the master mold. The solid red lines are fitted lines based on the experimental data.

A test structure analysis was performed on the microchannels, as shown in Figure 6a, to investigate the reliability of the proposed fabrication method. Further, we aimed to find the relationship between the resulting sizes of the collagen test microchannels vs. their corresponding PDMS mold replica sizes. Collagen usually shrinks during its fabrication process. Therefore, analyzing the test structures helped us quantify how shrinkage affects the shape of the final embedded microchannels.

Figure 6b,c show the resulting width and height of the fabricated collagen-based microchannels (W_Collagen_ and H_Collagen_) for a variety of sizes vs. the respective width and height of the corresponding molds (W_Mold_ and H_Mold_). These data helped us to determine the shrinkage behavior of the collagen microchannels containing a 2D film and a 3D porous scaffold.

As shown in Figure 6b, the fitted line (red line) almost matches the diagonal line y=x  (i.e., the W_Collagen_ values are similar to W_Mold_, implying that there is not a significant width-wise shrinkage).

The slope of the fitted red line representing the relationship between H_Collagen_ and H_Mold_ is less than unity (Figure 6c). We attributed this to the shrinkage of the collagen-based microchannels during the freeze-drying process. Based on the slope of the plot in Figure 6c, a decent approximation for $H_{\mathrm{Collagen}}$ can be obtained by multiplying the $H_{\mathrm{Mold}}$ by a correction factor of 0.8.

The perimeter can be considered as an indicator of the effective surface area of the microchannel. This interior surface area can be used to determine the effective surface area for cell culturing inside the collagen-based microchannels. The overall shrinkage for the microchannels was also quantified based on the percentage of reduction in the $P_{\mathrm{Collagen}}$ values compared to $P_{\mathrm{Mold}}$ values. As shown in Figure 6d, the perimeters of the collagen scaffold microchannels are less than the corresponding perimeter of the molds. The shrinkage of the fabricated microchannels was calculated based on the percentage of change in the size of the collagen channels’ perimeters compared to their corresponding replica mold perimeters. Figure 6e shows that the perimeter of the microchannels shrunk by between 15 and 30% of their target size. The shrinkage of collagen depends on the protocol used for its preparation. According to Yeong et al., the shrinkage of freeze-dried collagen (using 1% collagen slurry and a freezing temperature of −20 °C) is about 17% [47]. Sachlos et al. reported the shrinkage of collagen scaffolds fabricated based on critical-point drying to be ~70% when collagen slurry with a concentration of 1% was used. This shrinkage value decreased to ~55% using collagen slurry with a higher collagen content (≥2% *w*/*v*) [48].

The permeability of the 3D collagen scaffold and 2D collagen films were measured separately to evaluate the permeability of different components used in the collagen microchannels shown in Figure 6a. The permeability of the 3D porous collagen scaffold is important as it affects cell and chemical penetration into the scaffold after culturing. Here, we assessed how a 2D collagen film lining on the inner wall of a microchannel embedded inside the collagen scaffold affects the penetration of flowing media into the 3D porous collagen scaffold. Following the procedure explained in the Materials and Methods section, it took an average time of 11.88 ± 7.2 (n = 3) minutes for 2 mL of water to pass through the 3D porous collagen scaffold without a 2D lining. Assuming a constant flow rate during the experiment, the permeability of our 3D collagen scaffold was calculated to be $7.04\times 10^{-12}\pm 4.5\times 10^{-12}{\;m}^{2}$ based on Darcy’s equation [49]. The permeability of the 3D porous collagen lined with the 2D collagen film was measured to be zero as no water was passed through the collagen film when a similar experiment was implemented. This was expected as the 2D film is not porous and acts as a barrier to the convective flow from the channel into the porous collagen.

### 3.4. Fabrication of Embedded Microchannels within the Collagen Scaffold

**Figure 7:**
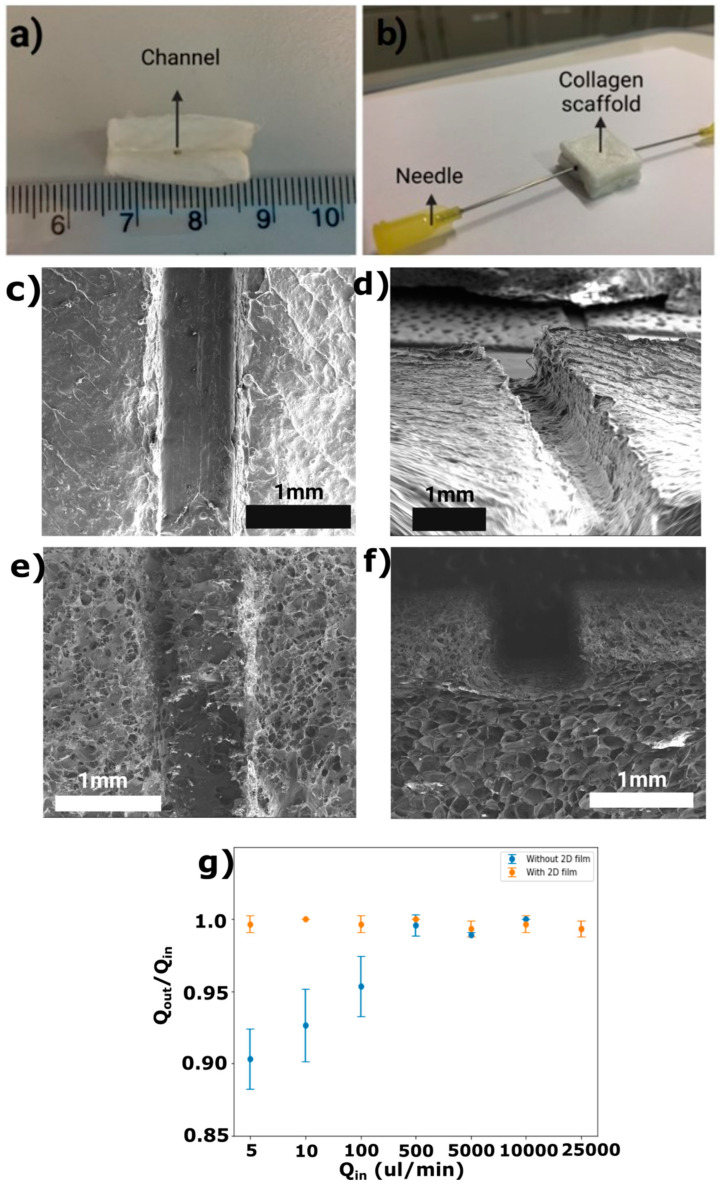
Fabricated enclosed microchannels: (**a**) cross-sectional view of the final collagen scaffold; (**b**) final collagen microchannel with embedded needles as inputs and outputs; (**c**) top-view and (**d**) side-view SEM images of the fabricated collagen scaffold containing microchannels based on the integration of 2D collagen film and a 3D scaffold; (**e**) top-view and (**f**) side-view SEM images of the fabricated collagen scaffold containing microchannels without the 2D collagen film; (**g**) the ratio of outflow rates to inflow rates with different flow rates when the 2D collagen membrane was used (shown in orange) and when no 2D BM was used (shown in blue).

Here, we utilized the bonding method shown in Figure 2 to fabricate a collagen-based microchannel containing 3D porous collagen and a 2D film (see Figure 7a). Two needles were then used at the inlet and outlet of the channels in order to allow media to flow through the scaffolds (see Figure 7b). We fabricated two types of collagen-based microchannels: (i) a microchannel based on the integration of 2D collagen film and a 3D porous scaffold (see Figure 7c,d) and (ii) a microchannel based on only the 3D porous collagen scaffold without the 2D film integration (see Figure 7e,f). Based on the SEM images in Figure 7c,d, the surface of collagen-based microchannels with 2D films does not have any pores. However, SEM images of these microchannels based on 3D porous collagen scaffolds reveal a porous structure of the microchannel walls when no 2D film is used during microfabrication.

A leak test was performed to assess the potential application of the proposed collagen-based enclosed microchannel for future use as a blood vessel. To evaluate the dynamic similarity of the fluid flow inside different microchannels, the Reynold’s number $\left(Re=\frac{U_{m}D_{h}}{\nu }\right)$ was calculated, where ν is the kinematic viscosity of the media, $U_{m}$ is the average velocity of the flowing media, and $D_{h}$ is the hydrodynamic diameter. In the literature, a wide range of Reynold’s numbers have been reported for veins. For example, Jayalalitha et al. [50] reported a Reynold’s number of 7.28, but a higher value of Re = 150 was reported by Ostadfar [51]. Furthermore, the Reynold’s number for blood flow in arteries has been reported to be between 110 and 850 [51]. In our experiments, a flow rate of 5 mL/min (with a corresponding Reynold’s number of 110) could result in a similar dynamic condition as the flow of blood in veins. Also, flow rate values of 10 and 25 mL/min with corresponding Reynold’s number values of 226 and 567 can simulate the flow of blood in the arteries. We used this wide range of flow rates to test the durability of our collagen microchannels. The result of the leak test for the final fabricated collagen-based microchannel is shown in Figure 7g. When a 2D collagen scaffold was used during microfabrication to line the microchannel, the outflow rates ($Q_{out})$ were the same as the corresponding inflow rates ($Q_{in})$ regardless of the flow rate or residence time (i.e., the total time that a fluid particle spends inside the channel from the inlet to the outlet, found by dividing the channel length by the fluid average velocity). This can be attributed to the existence of the non-permeable 2D collagen film.

To investigate the effect of using 2D collagen film, we repeated this leak test for the situation where the microchannel was fabricated without collagen film. In this situation, it was hypothesized that some of the flowing media would penetrate through the porous structure of the 3D collagen scaffold. The results showed that at large flow rates, the inflow and outflow rates were equal. However, when smaller flow rates were tested, the outflow rate values were not equal to the inflow rate values (see Figure 7g). This is because when the flow rates are relatively low, the average residence time of the media in the channel is relatively large. As a result, a larger portion of water can penetrate into the porous scaffold. The flow rates of penetrated water into the porous collagen were 0.48 ± 0.1, 0.73 ± 0.25, and 4.67 ± 2.08 µL/min for the corresponding inflow rates of 5, 10, and 100 µL/min, respectively. For the higher inflow rates, the penetrated flow rates of the fluid into the porous collagen could not be measured accurately. More accurate methods for quantifying flow rates should be used in the future to obtain the penetration flow rates appropriately.

## 4. Conclusions

A novel method is introduced here for fabricating a collagen-based scaffold with embedded enclosed microchannels that offers several advantages over the conventional techniques. Previous microfabrication techniques have mainly been based on polymer-based systems, which need surface modification to enable the anchorage of the cells. However, our fabricated scaffold is completely made of natural biomimetic collagen. Therefore, our collagen-based scaffold may serve as a more biologically relevant and more efficient biocompatible co-culture system for disease studies and drug discovery. To the best of our knowledge, this is the first study where enclosed microchannels are fabricated in a 3D porous collagen scaffold containing an integrated 2D collagen film. The 2D layer simulates the structure of BMs in natural tissues, which preserves the integrity of channels while inhibiting leakage of flowing medium through the microchannel. The impermeability of the 2D collagen film makes it an ideal candidate as a BM analog for larger blood vessels such as veins and arteries, wherein the extent of the permeability is determined by the cultured cells rather than the corresponding BMs. Sacrificial micro- and nano-particles can be integrated into the 2D collagen film in the future, where their post-fabrication removal could result in a controlled production of micro- and nano-pores in this layer. In the future, the scaffold must be crosslinked to improve its mechanical properties and further tests must be conducted to evaluate its dimensional stability and the flow within the microchannel, especially at more biologically relevant temperatures. The proposed integrated enclosed microchannel can be used to test cells in a dynamic model under a specific flow rate simulating the flow of blood in vessels. In future studies, endothelial cells will be cultured on collagen microchannels in order to study the interaction of blood cells with the surrounding engineered tissues.

## Data Availability

The data presented in this study are available on request from the corresponding author.

## Supplementary Materials

The following supporting information can be downloaded at https://www.mdpi.com/article/10.3390/mi15081031/s1: Figure S1. Steps required to obtain the average width of the collagen-based microchannels. (a) the optical image from the cross-sectional view of a collagen channel, (b) the edges of the microchannel are detected, (c) the edges corresponding to the side walls of the microchannel are kept, and the rest of the edges in the image are removed, and (d) horizontal lines are drawn by the MATLAB routine, and the mean of their lengths is determined. Figure S2. Steps required to obtain the average height of the collagen-based microchannels: (a) the optical image from the cross-sectional view of the collagen channel, (b) the edges of the microchannel are detected, and the baseline from which the height is calculated is added to the image of edges by the user, (c) the edges corresponding to the baseline and the bottom of the channel is kept, and the rest of edges in the image are removed, and (d) vertical lines are drawn by the MATLAB routine, and the mean of their length is determined. Figure S3. Image analysis to obtain the thickness of the 2D collagen film, (a) an optical image obtained from the side view of a 2D collagen film, and (b) vertical lines which were drawn between the boundaries of the 2D layer whose corresponding length mean represents the 2D film thickness. Figure S4. Edge detection and perimeter calculation using image analysis: (a) the cross-sectional view of the original image and (b) the edges of the microchannel walls (shown in blue) and the connecting lines (shown in green) between the subsequent edge points. Figure S5. Channel area determination using image analysis: (a) cross-sectional view of a 3D/2D integrated collagen scaffold with an embedded microchannel, and (b) the filtered image whose number of white pixels corresponds to the cross-sectional area of the collagen microchannel. Figure S6. Leak test for evaluating the performance of the fabricated microchannels: (a) the schematic representation of the leak test, (b) the actual collagen scaffold used for the leak test with the inlet and outlet, (c) the collagen scaffold comprising a channel lined with 2D collagen film, and (d) the scaffold comprising a channel without 2D collagen film. Panels a, c, and d are created with BioRender.com. Reference [34] is cited in the Supplementary Materials.
