# Supplementary material for: Fabrication of Porous Collagen Scaffolds Containing Embedded Channels with Collagen Membrane Linings
Source: Micromachines (Basel). 2024 Aug 14;15(8):1031. doi: 10.3390/mi15081031 (PMC11356104; doi:10.3390/mi15081031)
Supplement: Supplementary file 1 [file micromachines-15-01031-s001.zip › micromachines-3130907-supplementary.pdf]

Supplementary Information File

Fabrication of Porous Collagen Scaffolds Containing Embedded Channels with Collagen Membrane Linings

Neda Fakhri, Arezoo Khalili, Terry Sachlos*, Pouya Rezai*

Department of Mechanical Engineering, York University, Toronto, ON, CANADA

* Corresponding Authors: 4700 Keele St, Toronto, ON, M3J 1P3, Canada; Tel: 416-736-2100 ext. 44703; Emails: prezai@yorku.ca; sachlos@lyorku.ca

Image Processing

To obtain the pore size distribution and the mean pore size, we used a MATLAB code developed by Rabbani et al.[1]. Another MATLAB code was developed to calculate the width of the corresponding collagen-based test structures (**Figure S1a**).

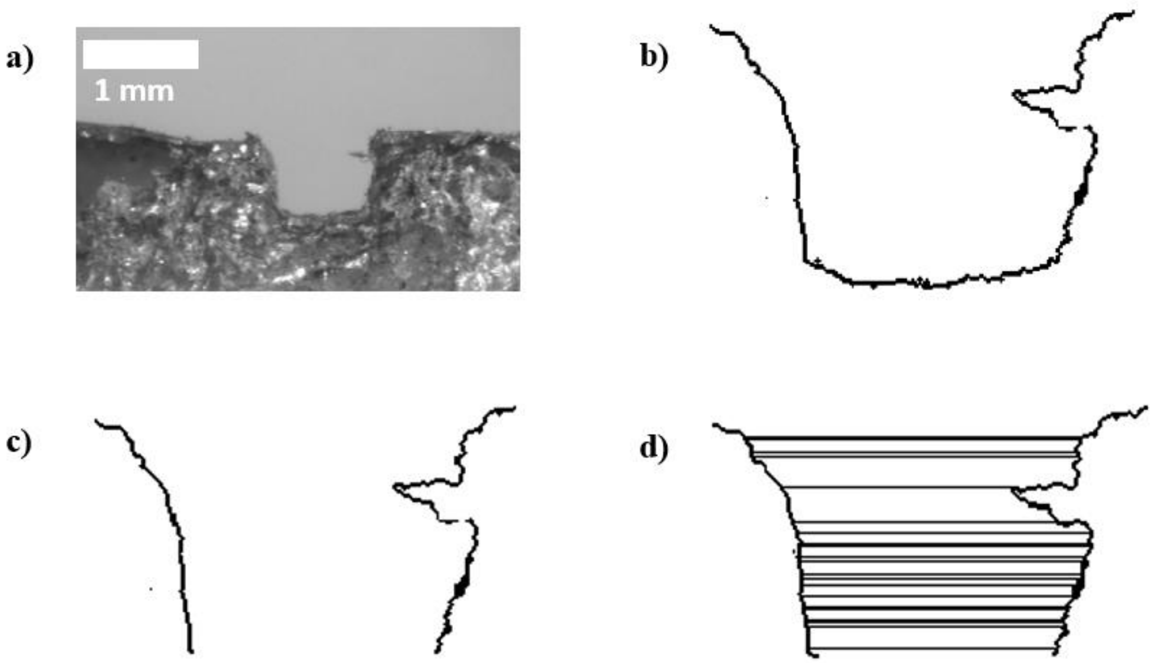

Figure S1. Steps required to obtain the average width of the collagen-based microchannels. a) the optical image from the cross-sectional view of a collagen channel, b) the edges of the microchannel are detected, c) the edges corresponding to the side walls of the microchannel

are kept, and the rest of the edges in the image are removed, and d) horizontal lines are drawn by the MATLAB routine, and the mean of their lengths is determined.

To do so, the built-in MATLAB function “edge.m” was used for edge detection (see **Figure S1b**). Then, the edges corresponding to the side walls of the microchannels were selected by the user, and the remaining edges corresponding to the bottom of the microchannel were removed (see **Figure S1c**). Finally, a sufficient number of horizontal lines were drawn by the developed code between the detected edges of vertical microchannel walls (see **Figure S1d**), and their average length was reported as the width of the microchannel.

A similar procedure was followed to determine the height of the microchannels (see **Figure S2**). The additional required step was to add a baseline from which the height of the microchannels could be calculated.

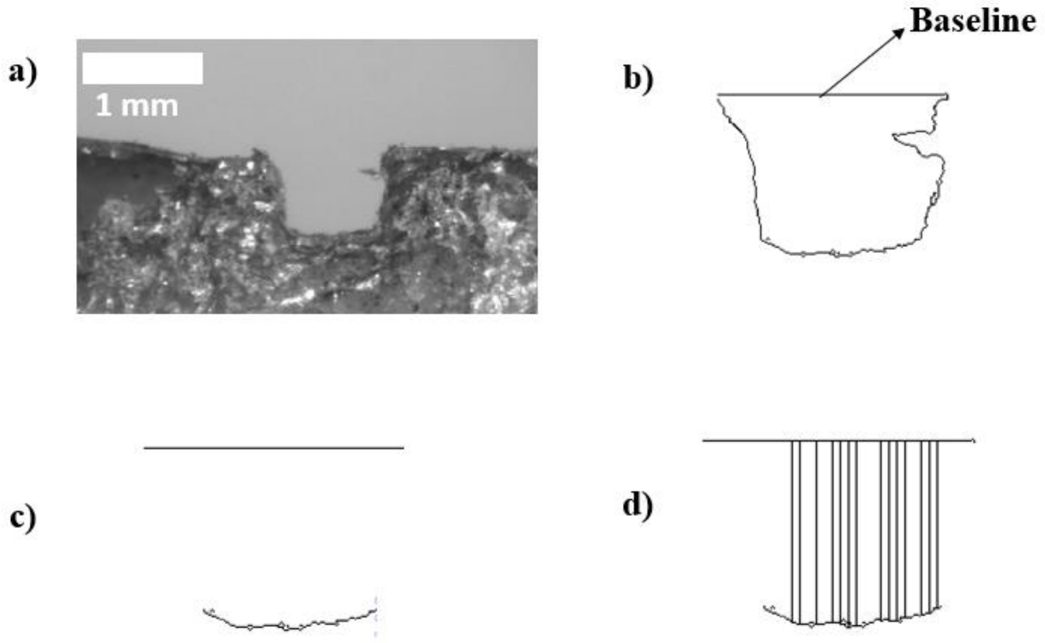

Figure S2. Steps required to obtain the average height of the collagen-based microchannels: a) the optical image from the cross-sectional view of the collagen channel, b) the edges of the microchannel are detected, and the baseline from which the height is calculated is added to the image of edges by the user, c) the edges corresponding to the baseline and the bottom of the channel is kept, and the rest of edges in the image are removed, and d) vertical lines are drawn by the MATLAB routine, and the mean of their length is determined.

The thickness of the 2D collagen films was also determined by a similar method as the height determination algorithm, whose associated steps were shown in Figure S4, except that a baseline is not required to be specified (see **Figure S3**). The average thickness of the collagen fibers (i.e., pore walls) was obtained using ten different pore walls from each of the SEM images in Figure 4.

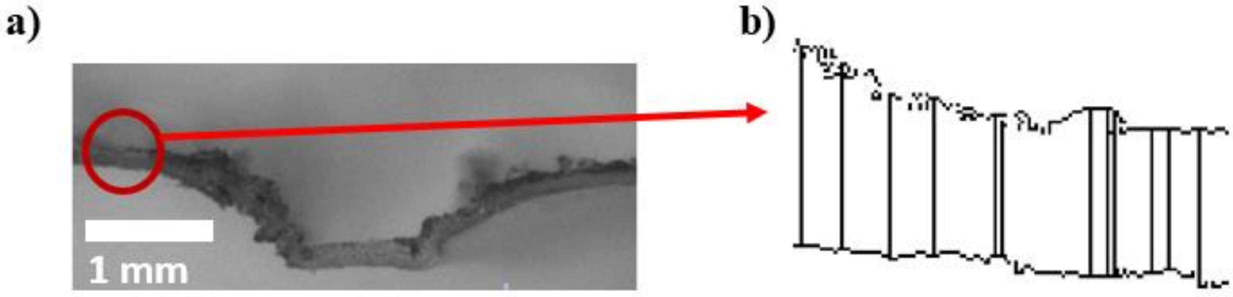

Figure S3. Image analysis to obtain the thickness of the 2D collagen film, a) an optical image obtained from the side view of a 2D collagen film, and b) vertical lines which were drawn between the boundaries of the 2D layer whose corresponding length mean represents the 2D film thickness

Another image analysis algorithm was used to obtain the perimeter of the channels (see **Figure S4a**). In this algorithm, the edges of the microchannels are initially detected. Next, the MATLAB-developed algorithm determines some representative points from the subsequent edge points. Afterward, the perimeter is approximated by adding the lengths of all connecting lines passing through the representative points (i.e., green lines in **Figure S4b**).

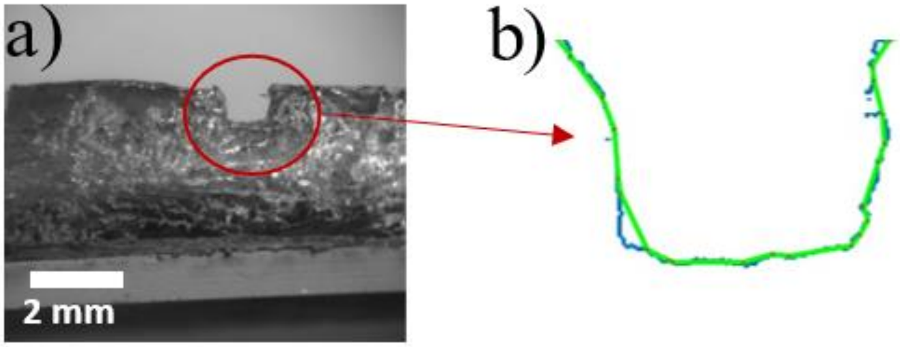

Figure S4. Edge detection and perimeter calculation using image analysis: a) the cross-sectional view of the original image and b) the edges of the microchannel walls (shown in blue) and the connecting lines (shown in green) between the subsequent edge points.

The cross-sectional area for the final collagen-based microchannels was also obtained using image analysis. As shown in **Figure S5a,** the image containing a cross-sectional view of the channels was converted into a binary image. The number of resulting white pixels (see **Figure S5b**) in the binary image is proportional to the cross-sectional area and can be obtained by multiplying the number of pixels by the area-to-pixel ratio.

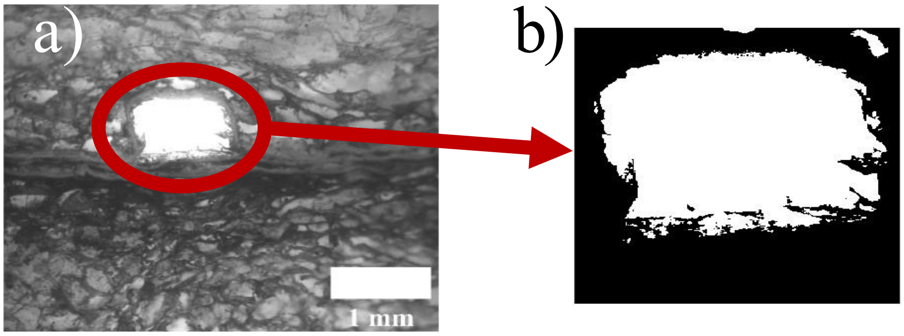

Figure S5. Channel area determination using image analysis: a) cross-sectional view of a 3D/2D integrated collagen scaffold with an embedded microchannel, and b) the filtered image whose number of white pixels corresponds to the cross-sectional area of the collagen microchannel.

Leak Test

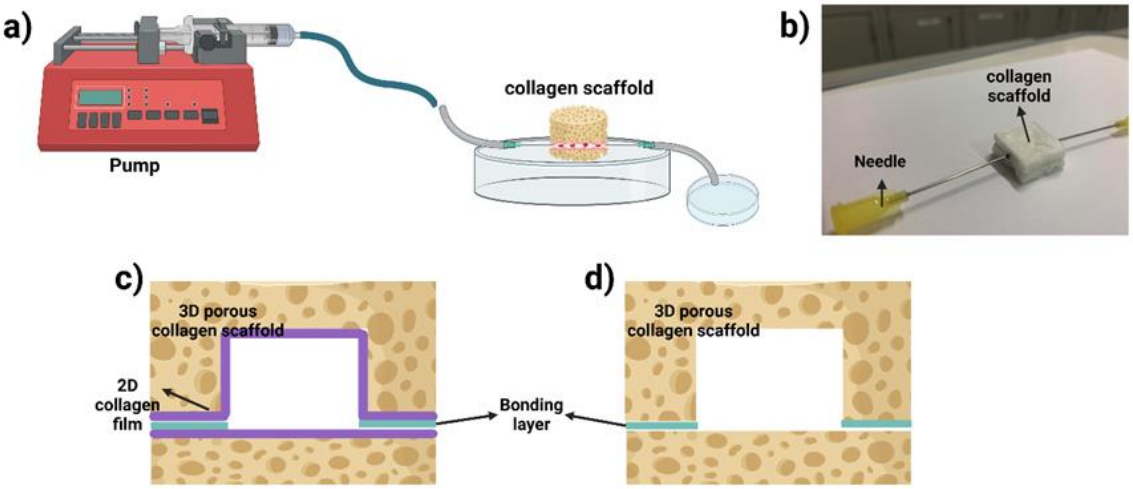

Figure S6. Leak test for evaluating the performance of the fabricated microchannels: a) the schematic representation of the leak test, b) the actual collagen scaffold used for the leak test with the inlet and outlet, c) the collagen scaffold comprising a channel lined with 2D collagen film, and d) the scaffold comprising a channel without 2D collagen film. Panels a, c, and d are created with BioRender.com.

1. Rabbani A, Salehi S. Dynamic modeling of the formation damage and mud cake deposition using filtration theories coupled with SEM image processing. *Journal of Natural Gas Science and Engineering.* 2017;42:157-168.
